# *¡Míranos!* a comprehensive preschool obesity prevention programme in low-income Latino children: 1-year results of a clustered randomised controlled trial

**DOI:** 10.1017/S1368980022002439

**Published:** 2022-11-11

**Authors:** Zenong Yin, Yuanyuan Liang, Jeffrey T Howard, Vanessa Errisuriz, Vanessa Marie Estrada, Cristina Martinez, Shiyu Li, Sarah Lynn Ullevig, Erica Sosa, Todd Olmstead, Sharon Small, Dianne Stanton Ward, Deborah Parra-Medina

**Affiliations:** 1 The University of Texas at San Antonio, Department of Public Health, HCaP, San Antonio, TX, USA; 2 The University of Maryland, School of Medicine, Department of Epidemiology and Public Health, Baltimore, MD, USA; 3 The University of Texas at Austin, Latino Research Institute, Austin, TX 78712, USA; 4 UT Health San Antonio, School of Nursing, San Antonio, TX, USA; 5 The University of Texas at Austin, LBJ School of Public Affairs, TX, USA; 6 Parent/Child Incorporated of San Antonio and Bexar County, San Antonio, TX, USA; 7 Department of Nutrition, Gillings School of Global Public Health, University of North Carolina at Chapel Hill, NC, USA

**Keywords:** Energy balance-related behaviour, Latino children, Physical activity, Nutrition policy, Childcare

## Abstract

**Objective:**

To test a culturally tailored obesity prevention intervention in low-income, minority preschool age children.

**Design:**

A three-group clustered randomised controlled trial.

**Setting:**

Twelve Head Start centres were randomly assigned to a centre-based intervention, a combined centre- and home-based intervention, or control using a 1:1:1 ratio. The centre-based intervention modified centre physical activity and nutrition policies, staff practices, and child behaviours, while the home-based intervention supported parents for obesity prevention at home.

**Study outcomes:**

The primary end point was change in children’s BMI (kg/m^2^) at post-test immediately following completion of the 8-month intervention. Secondary end points included standardised scores for BMI (BMIz) and body weight (WAZ), and BMI percentiles (BMI pctl).

**Participants:**

Three-year-old children enrolled in Head Start in San Antonio, Texas, with written parent consent (*n* 325), 87 % Latino, 57 % female with mean age (sd) of 3·58 years (0·29).

**Results:**

Change in BMI at post-test was 1·28 (0·97), 1·28 (0·87) and 1·41 (0·71) in the centre + home-based intervention, centre-based intervention and control, respectively. There was no significant difference in BMI change between centre + home-based intervention and control or centre-based intervention and control at post-test. BMIz (adjusted difference –0·12 (95 % CI, –0·24, 0·01), *P* = 0·06) and WAZ (adjusted difference, –0·09 (–0·17, –0·002), *P* = 0·04) were reduced for children in centre + home-based intervention compared with control group.

**Conclusions:**

There was no reduction in BMI at post-test in children who received the intervention. Findings shed light on methodological challenges in childhood obesity research and offer future directions to explore health equity-oriented obesity prevention.

National data in the USA demonstrate that obesity (BMI > 95th percentile for age and gender) prevalence among children aged 2–18 years has trended upwards from 1963–1965 to 2017–2018^(1)^ and disproportionally affected children from a minority background and low-income families^(2)^. In 2017–2018, obesity affected 17·3 % of Latino (i.e. Mexican-American) children compared with 12·4 % of White children between 2 and 5 years old^(3)^. Obesity in young children raises the risk for cardiometabolic, psychological and psychosocial disorders, developmental delays, as well as healthcare costs across the lifespan^(4)^. Early onset of obesity is linked to increased exposure to an obesogenic environment, characterised by a lack of access to resources or support to regulate energy balance-related behaviours (EBRB; i.e. physical activity (PA), sedentary behaviours, sleep and dietary habits)^(5)^, which also disparately burdens children from low-income and minority families^(6)^. Successful management of EBRB can reverse the trend of positive energy balance, especially among those children most at-risk for obesity^(5)^.

Since the first national call to combat early childhood obesity in the USA in 2011^(4)^, obesity prevention interventions for young children aged 3–5 years have produced mixed results on obesity outcomes with less than half of interventions improving weight-based outcomes and successful interventions demonstrating small effect sizes with low-quality evidence^(7,8)^. These lackluster results were confirmed by two recent reviews commissioned by the WHO that found small effects on weight or body composition measures as well as EBRB in ‘effective’ randomised controlled trials (RCT) in children aged 2–5 years^(7)^. Implementation of evidence-based guidelines and policies in childcare settings has demonstrated promising but small reductions in obesity in disadvantaged young children^(7–9)^. The limited impact of interventions addressing policies in childcare settings may be due to the failure to address the influence of parental behaviours and home life on obesity. As such, research has called for greater family engagement in obesity interventions to reduce the influence of obesogenic parental practices and home environments on obesity. Given the complexity of the causes of obesity, an emerging body of literature points to promising multi-level^(8,10)^ and multi-behaviour^(11)^ approaches to address the various challenges young children from low-income minority families face in childcare settings^(8)^. Few studies have tested the efficacy of multi-level and multi-behaviour approaches in developed and developing countries. Furthermore, there is no evidence demonstrating whether combining activities targeting the centre and home can enhance the impact of obesity prevention programmes in young children, especially those from low-income Latino families^(12)^.

Head Start is a federally funded programme that offers services and supports in school readiness, health, nutrition, and family well-being to children aged 0 to 5 years from low-income families in the USA^(13)^. In 2018, 16·6 % of children attending Head Start in Texas were classified/met criteria for/had obesity^(14)^, higher than the national prevalence of 13·7 % in children of the same age^(3)^. To explore the feasibility of obesity prevention in early childcare settings, we conducted a pilot study in collaboration with local agencies administering the Head Start programme to identify strategies to address barriers and facilitators of programme implementation, such as PA and nutrition policies, education and training of staff and parents, and developmentally and culturally appropriate activities underpinning the obesogenic environment in childcare and home settings^(15)^. Based on findings from our earlier pilot work^(15)^, we refined and tested ‘*¡Míranos!* Look at Us, We Are Healthy! (*¡Míranos!)*’, a culturally tailored obesity prevention programme for low-income, primarily Latino children enrolled in Head Start in San Antonio, Texas. *¡Míranos!* modified centre PA and nutrition policies, staff practices, and child EBRB in the centre-based intervention (centre-based intervention), while the home-based intervention offered training and support to parents for obesity prevention at home. This article reports findings on the study’s primary outcome, change in BMI, at the end of the 8 months of *¡Míranos!* intervention. We hypothesised that, compared with children in the control group, children who received the combined centre- and home-based intervention (centre + home-based intervention) or the centre-based intervention alone would have significantly smaller increases in BMI at post-test. The intervention’s effect on sex- and age-standardised BMI and body weight was also examined.

## Methods

### Study design

*¡Míranos!* was a three-arm clustered RCT efficacy study conducted at twelve Head Start childcare centres that were administered by two social service agencies in San Antonio, Texas. Both agencies had a long history of offering early childhood education to low-income families and were involved from the early stages of *¡Míranos!* intervention development from 2009 to 2011. The *¡Míranos!* research team included the Head Start senior administrators, food service director, study investigators, study intervention specialists and study staff. Study preparation and administration were conducted jointly by Head Start and academic research staff. During the first year of the study, the research team held monthly meetings to develop the study protocol, plan the implementation and discuss logistics and challenges; in the following years, bi-monthly team meetings were conducted. Before conducting the RCT, the research protocol was piloted at two Head Start centres to refine and increase the feasibility and acceptability of the intervention, assessments and process evaluation. Study outcomes were assessed at baseline before the commencement of the intervention, post-test immediately following the 8-month intervention (post-test) and follow-up assessment at month 21 (follow-up). The primary end point of the study was the change in BMI between the centre + home-based intervention and control, and between the centre-based intervention and control, at the end of the 8-month *¡Míranos!* intervention.

### Study setting and participants

Two local social service agencies operated forty-nine Head Start centres serving low-income families that met the federally defined Head Start eligibility criteria (i.e. poverty level adjusted for family size). Over 85 % of the children enrolled in the Head Start centres were Latino/Hispanic. Study eligibility criteria for Head Start centres were as follows:(1) enrolment of ≥ 75 % of children identified as Latino, (2) willingness to receive treatment randomisation, (3) on-site access to an outdoor playground, (4) serve meals from the study central kitchen and (5) agreement to not participate in new health-related studies during the study period. Disqualification of the centres was primarily due to not serving meals from the central kitchen used in the study. Eligibility criteria for child participants included (1) 3-year-old at the beginning of the school year, (2) enrolment in a 3-year-old-only classroom and (3) one child per family. If more than one child from a family was identified, only the first child from the family encountered by the data collection staff was included in the study. A parent/guardian signed an informed consent form for their child’s study participation. Two cohorts were recruited (Cohort 1: August 2018–May 2020; Cohort 2: August 2019–May 2021). Due to the COVID-19 pandemic, intervention delivery and data collection were disrupted in spring 2020. Therefore, we report findings for Cohort 1 children who completed the 8-month intervention and had outcome assessment data for either baseline or post-test.

### Randomisation and concealment

Twelve Head Start centres that had the highest enrolment of 3-year-old children and met study eligibility criteria were randomly assigned to the combined centre + home-based intervention, the centre-based intervention or the control group (control) in a 1:1:1 ratio. Treatment randomisation was generated by the study biostatistician using R version 3.3.2 (R Development Core Team, Austria) with stratification by Head Start agency (agency one *v*. agency two) and centre size (small (≤ two 3-year-old classrooms) *v*. large (≥ three 3-year-old classrooms)) for equal representation. Immediately after completing the baseline assessment, one Head Start centre was closed due to an unanticipated organisational restructure. One centre from the same agency replaced the closed centre.

All participants were blinded to treatment conditions until the completion of baseline assessments. Head Start and research staff, including data collectors, were not blinded to treatment conditions. The intervention was implemented by trained Head Start staff and parent peer educators with technical assistance from the study’s intervention specialists who did not participate in intervention activities. The research staff did not deliver intervention activities.

### The *¡Míranos!* intervention

The rationale and details of the *¡Míranos!* intervention are reported elsewhere^(16)^. Briefly, *¡Míranos!* employed evidence-based strategies to promote key messages (Table 1) targeting children’s EBRB at the childcare centre and home and was based on the socioecological model (targeting multiple levels of influence, i.e. children, centre policies and practices, and home environment)^(17)^, child developmental theory (role modelling, and offering age-appropriate and culturally appropriate activities)^(18,19)^, and social learning theory (increasing efficacy of Head Start staff and parents in applying evidence-based strategies via training and support)^(20)^. The intervention activities were tailored to meet the cultural, linguistic, and logistical needs of Head Start parents and operators by incorporating values and norms relevant to Latino populations and accommodating Head Start’s organisational infrastructure^(16)^ (Supplement Table 1).

**Table 1:** *¡Míranos!* intervention key messages*

PA and nutrition policies	1. Educate children to develop healtdy habits for life 2. Offer 90 minutes free and teacher-led physical activity to children at tde centre every day 3. Offer balanced healtdy meals and snacks utilising tde USDA Child and Adult Care Food Program best practice recommendations
Staff	1. Be part of children’s play 2. Role-model healthy behaviours to children at all times 3. Be physically active for 30 minutes every day 4. Eat healthy MyPlate meals every day
Parents	1. Help your child get 30 to 60 min of physical activity at home every day 2. Serve fruits and vegetables to your child at every meal 3. Limit your child’s TV watching to less than 2 h every day 4. Avoid offering sugar-added beverages to your child 5. Turn the TV off during meals 6. Help your child get at least 10 h of sleep every day

* Justification for the selection of the key messages is discussed elsewhere^(16)^.

We tailored the *¡Míranos!* home-based intervention to increase parent participation and engagement by: (1) offering parent education during child pick-up to reduce parent time/transportation burden; (2) using bilingual parent-peer-educators to enhance communication and trust; (3) using visual and bilingual displays to reduce language barrier; (4) portraying images of Latino children and families; (5) addressing barriers (e.g. access to PA and healthy food, hot summers) and enablers (e.g. social support from family (familismo)) commonly cited by Latino families; and (6) offering tangible incentives. Based on feedback from parents in our pilots, we have highlighted healthy foods common among Latinos in San Antonio, using inexpensive ingredients that are available locally, and identified culturally appropriate activities and ways for parents to meet expert recommendations (e.g. how to keep a child busy while limiting screen time).^(15)^

The centre-based intervention in *¡Míranos!* included modification of centre PA and nutrition policies, modified meal patterns, enhanced PA and gross motor programme, supplemental classroom health education for children, and a voluntary staff wellness programme. The centre-based intervention was designed to address key barriers to obesity prevention in childcare settings identified by childhood obesity experts^(9)^. The home-based intervention consisted of monthly peer-led obesity prevention parent education sessions and take-home bags, family newsletters containing healthy recipes and community PA and nutrition resources, family health challenges, and three home visits conducted by Head Start family service workers. Head Start centre directors recruited two to four parents from their centre to serve as peer educators to deliver education sessions. Preference was given to parents who spoke English and Spanish and had a history of volunteering at a centre. Parent peer educators received a small stipend (up to $240) to compensate for their participation in training and delivery of sessions. Trained peer educators delivered eight monthly education sessions. Education sessions were held in a designated hallway or room at each centre during child pick-up time and lasted 15–20 min. Parent peer educators used wall posters and live demonstrations to promote expert recommendations and evidence-based strategies related to child EBRB. During education sessions, parents were also given a scavenger hunt with six questions to answer by viewing the posters and talking with peer educators, who provided instant feedback and social support for managing EBRB at home. Head Start family service workers also incorporated *¡Míranos!* activities in their home visits with parents, working with parents to review various health topics and help them set goals and develop an action plan to achieve those goals to make the home environment more conducive for healthy behaviours. Activities in the centre + home-based intervention were synchronised so that children were exposed to the same messages at the centre and at home. Intervention activities were implemented in 3-year-old classrooms only and followed a pre-established schedule over the 8-month intervention period

Before the start of *¡Míranos!*, Head Start centre staff (i.e. centre director, teachers, teacher assistants, family service workers and food service workers), central kitchen workers and senior curricular staff completed 11 to 15 h of training developed by the research team, including two half-day in-person training sessions^(16)^. All were compensated for their time.

Control centres implemented the Head Start-endorsed PA and nutrition programme, ‘I Am Moving, I Am Learning’^(21)^. Parents of children in control centres were invited to participate in a six-session, nutrition-themed literacy education programme supported by a local grocery chain. Instead of using a classic ‘no treatment’ control, we used an active control to offer attention and some benefits to enhance the buy-in and retention of study participants.

### Outcome measurement

#### Children’s weight and height

Weight (with light clothing) was measured in kilograms, and height (without shoes) was measured in centimetres. The research staff made concerted efforts to schedule the weight and height measurement session during the early morning hours. Each child’s height and weight were measured twice by a research staff member, and the average of the two measurements was used to calculate BMI (kg/m^2^). In cases where there was a discrepancy between two measurements (i.e. greater than 0·5 cm for height and 0·25 kg for weight), research staff recorded another measurement and an average of all three measurements was used to calculate BMI. A second research staff member was present and repeated height and weight measures for the first and every subsequent fifth child to ensure data accuracy and quality. The primary outcome of the study was a change in a child’s BMI from baseline to post-test, i.e. excessive weight gain that is used as a proxy of increased adiposity^(22)^. Standardised scores for BMI (BMIz), body weight (WAZ) and standardised percentiles for BMI (BMI %ile) based on the 2000 CDC Growth Charts^(23)^ were also calculated as additional adiposity measures^(24)^. We chose BMI as the primary outcome for its documented validity in children^(22)^, while BMIz and WAZ have also been shown to be strong predictors of obesity in young children^(24)^.

#### Demographic measures

Child and family demographic information and health history, including mother’s education level, parental marriage status, the language most spoken at home, child’s asthma status, and family history of diabetes, were collected from Head Start records and parents.

### Statistical analysis

Demographics and characteristics of Head Start centres and study participants were summarised using descriptive statistics and compared between the three groups using the *χ* ^2^ test or Fisher’s exact test for categorical variables and Kruskal–Wallis H test for continuous variables. The study hypothesis was tested based on the Intent-to-Treat principle^(16)^. For each outcome of interest (i.e. BMI, BMIz, BMI %ile and WAZ), we used a three-level (time nested within child and child nested within centre) linear mixed effects model to examine group differences with time (baseline *v*. Post-test), treatment group (centre-based intervention *v*. centre + home-based intervention *v*. control), the interaction between time and treatment group, and centre size as fixed predictors that were kept in the model regardless of statistical significance. Two random effects were included in the linear mixed effects model, one to account for the correlation among two measures nested within the same child and the other for the correlation among children nested within the same centre. Data were assumed to be missing at random. In the full linear mixed effects model, child’s age at baseline, age squared, gender, race/ethnicity, asthma, child’s height at baseline (quartiles), change in height (quartiles), mother’s education, language spoken most often at home, parent marital status and family history of diabetes were included as confounders that were associated with variation in body weight in children^(25)^. Baseline height in centimetres and change in height over time were categorised into four groups based on their quartiles: baseline height group (1 = 83–94·7; 2 = 94·8–97·6; 3 = 97·7–101·1; 4 = 101·2–116) and change in height group (1 = 1·35–3·6; 2 = 3·65–4·2; 3 = 4·25–4·75; 4 = 4·8–6·55). Height in quartiles and change in height group were also included as covariates to adjust for the rate of growth^(26)^. We employed backward model selection to remove one non-significant (*P* > 0·05) confounder at a time from the confounder list above, and Akaike’s information criterion and Bayesian information criterion (BIC) guided the model selection process to select the final reduced model. All analyses were performed using Stata/SE (version 16).

### Sample size and power calculation

The planned study sample included twelve Head Start centres, four centres per group, with an average of twenty-nine children per centre (*n* 444) at baseline to achieve 80 % power to detect a group difference of 0·53 in BMI change at the end of the intervention (i.e. mean change of –0·03 in the CBI group or the CBI + HBI group *v*. mean change of 0·5 in the control group) using a two-sided *t* test with a significance level of 5 %, an intraclass correlation of 0·003,and a sd of 1·147 (PASS Version 11).

## Results

### Study participants

**Fig. 1:**
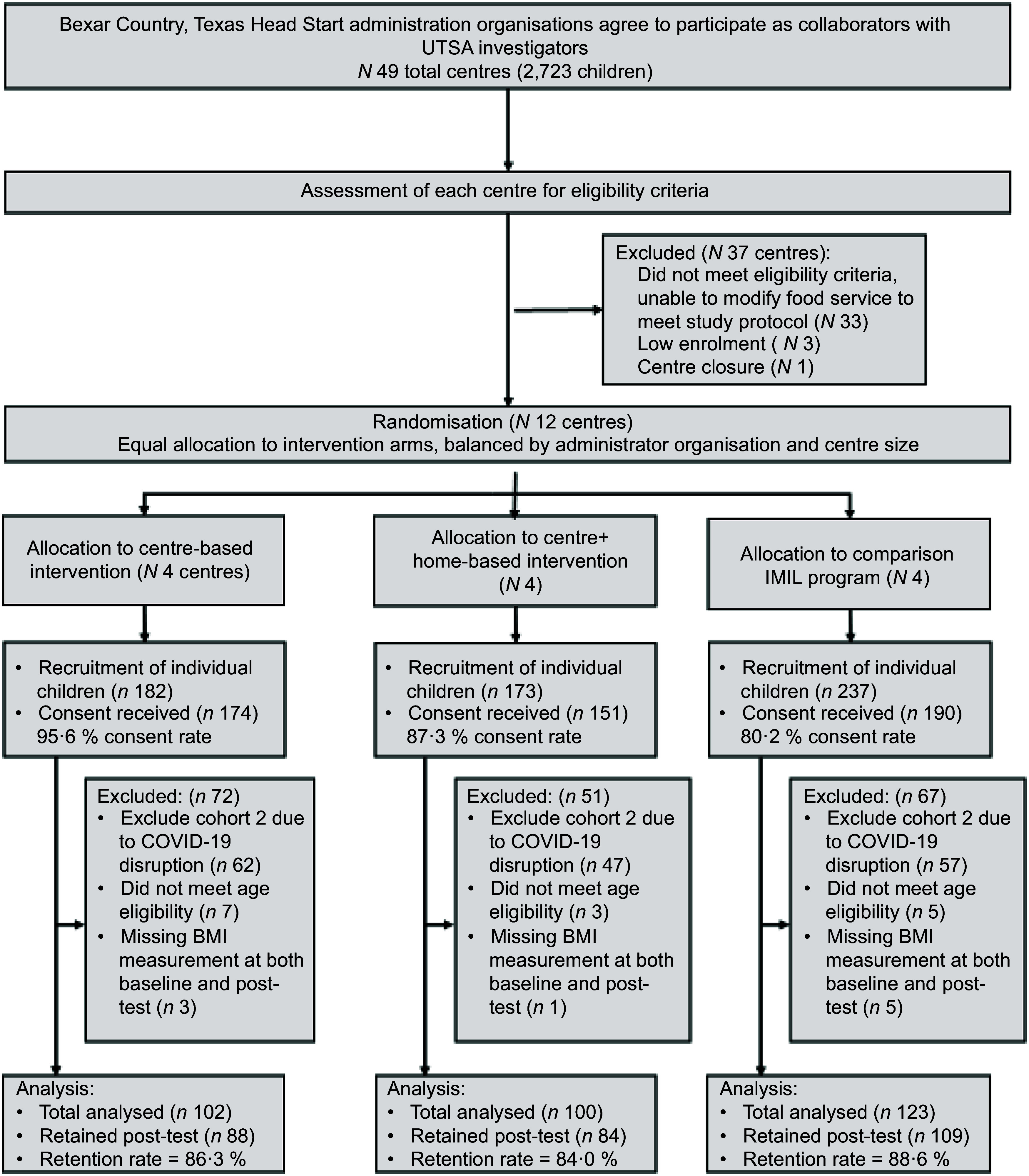
Data flow diagram

Table 2 displays the characteristics of the study participants who were primarily Latino/Hispanic. Overall parental consent rate was 87·0 % (*n* 515) for both cohorts. Cohort 2 children were excluded due to COVID-19 disruptions (*n* 166). Of 349 Cohort 1 children that consented, 93·1 % (*n* 325) completed the baseline assessment and 86·5 % were retained at post-test. The final analytic sample consisted of 325 children who had a valid BMI at baseline and post-test (100 centre + home-based intervention, 102 centre-based intervention and 123 control; Fig. 1). At baseline, the children’s mean (sd) age was 3·6 (0·3) years, 57 % were female, 87 % were Latino, 16·9 % had obesity, 13 % had a diagnosis of asthma and 41 % had a family history of diabetes. The majority of mothers reported completing high school or higher degrees (79 %), and more than half of children spoke English most often at home (56 %). There were no significant differences in children’s characteristics between the three groups, except that more of the centre-based intervention children were from small-sized centres.

**Table 2:** Baseline demographics and characteristics of Head Start centres and study participants

Variables	Total (*n* 325)		Centre + home-based intervention (*n* 100)		Centre-based intervention (*n* 102)		Control (*n* 123)		*P*-value
	*n*	%	*n*	%	*n*	%	*n*	%	
Centre data:									
Centre size									0·01
Small	203	63	65	65	73	72	65	53	
Large	122	38	35	35	29	28	58	47	
Child data:									
Child age at baseline, year									0·51
Median	3·6		3·6		3·6		3·6		
Q1, Q3	3·3, 3·8		3·4, 3·8		3·4, 3·8		3·3, 3·9		
Mean	3·6		3·6		3·6		3·6		
sd	0·3		0·3		0·3		0·3		
Child sex									0·12
Male	140	43	44	44	36	35	60	49	
Female	185	57	56	56	66	65	63	51	
Child race/ethnicity									0·11
Latino/Hispanic	281	86·46	82	82	86	84·31	113	91·87	
Non-Latino/Hispanic African American	21	7	7	7	10	9·8	4	3	
Other*	23	7	11	11	6	5·9	6	5	
Child with asthma	41	13	8	8	16	15·7	17	14	0·23
BMI category									0·93
Under/normal (BMI ≤ 85th percentile for age and gender)†	221	68	69	69	67	65·69	85	69·11	
Overweight (BMI between 85th and 94·9th percentile for age and gender)	49	15·08	13	13	17	16·67	19	15·45	
Obese (BMI ≥ 95th percentile for age and gender)	55	16·92	18	18	18	17·65	19	15·45	
Parent/family data:									
Mother education									0·76
Less than a high school degree	36	11	11	11	12	11·8	13	11	
High school degree/GED	143	44	45	45	40	39·2	58	47	
College or technical school degree	113	35	31	31	41	40·2	41	33	
N/A or missing	33	10	13	13	9	8·8	11	9	
Language spoken most often at home									0·63
English	181	56	58	58	62	60·8	61	50	
Spanish or other	77	24	22	22	24	23·5	31	25	
English and Spanish equally	46	14	13	13	11	10·8	22	18	
Not reported	21	7	7	7	5	4·9	9	7	
Parents not married	120	37	37	37	37	36·3	46	37	0·98
Family members with a history of diabetes	133	41	44	44	44	43·1	45	37	0·46

*P*-values are comparing the differences among the three groups (centre- and home-based intervention *v*. centre-based intervention *v*. control), categorical variables compared with the *χ* ^2^ test or Fisher’s exact test, and age compared using the Kruskal–Wallis H test.

* Other includes all non-Hispanics who are not African American.

† Due to a small number of children (*n* 10) in the category of underweight BMI < 5th percentile for age and gender, the underweight and normal weight categories were combined.

### Study outcomes

There was no significant difference in any of the unadjusted outcome variables across the three groups at baseline (e.g. baseline mean BMI (sd) = 16·76 (2·32), 16·6 (2), 16·54 (1·63) in the centre + home-based intervention, the centre-based intervention and control, respectively, *P* = 0·93; Table 3). In general, BMI declined, and body weight and height increased across all treatment groups from baseline to post-test, consistent with normal growth patterns of young children. Of note, control children had a significantly larger growth in height (mean change (sd) = 4·38 (0·88), *P* = 0·003) than children in the centre + home-based intervention (4·12 (0·79)) and the centre-based intervention (3·92 (0·87)).

**Table 3:** Descriptive statistics of outcomes of interest by study groups

Outcomes	Centre + home-based intervention (*n* 100)		Centre-based intervention (*n* 102)		Control (*n* 123)		Total (*n* 325)		*P*-value
	Mean	sd	Mean	sd	Mean	sd	Mean	sd	
Body weight									
Baseline	16·08	3·68	16·01	2·6	16·09	2·59	16·06	2·96	0·32
Post-test†	17·7	4·41	17·27	3·01	17·5	3·04	17·49	3·49	0·72
Change†	1·28	0·97	1·28	0·87	1·41	0·71	1·33	0·84	0·23
Height									
Baseline	97·5	5·35	98	4·31	98·39	4·77	97·99	4·82	0·28
Post-test†	102·05	5·85	101·93	4·57	102·85	5·04	102·32	5·16	0·52
Change†	4·12	0·79	3·92	0·87	4·38	0·88	4·16	0·87	0·003*
BMI									
Baseline	16·76	2·32	16·6	2	16·54	1·63	16·63	1·98	0·93
Post-test†	16·8	2·55	16·53	2·1	16·44	1·64	16·58	2·09	0·95
Change†	−0·15	0·72	−0·04	0·77	−0·07	0·53	−0·09	0·67	0·24
BMIz									
Baseline	0·61	1·31	0·56	1·24	0·57	1·13	0·58	1·22	0·95
Post-test†	0·67	1·34	0·58	1·21	0·61	1·08	0·62	1·2	0·96
Change†	−0·06	0·44	0·06	0·48	0·06	0·39	0·02	0·44	0·09
BMI %ile									
Baseline	63·34	30·22	63·21	29·14	64·97	27·62	63·91	28·84	0·95
Post-test†	64·88	30·34	64·79	29·83	67·19	26·67	65·75	28·72	0·96
Change†	−1	12·06	2·74	13·34	2·27	10·61	1·44	12·02	0·21
WAZ									
Baseline	0·18	1·3	0·3	1·21	0·29	1·07	0·26	1·19	0·28
Post-test†	0·24	1·36	0·25	1·26	0·31	1·09	0·27	1·22	0·7
Change†	−0·07	0·31	−0·03	0·33	0·02	0·26	−0·02	0·3	0·12

BMIz, BMI z-score; WAZ, body weight z-score.

Entries are mean (sd).

*P*-values are comparing the differences among the three groups based on the Kruskal–Wallis H test.

Change = post-intervention – baseline.

* *P* < 0·05.

† Sample sizes are 84, 88 and 109 in the centre- + home-based intervention, the centre-based intervention and control groups, respectively.

**Fig. 2:**
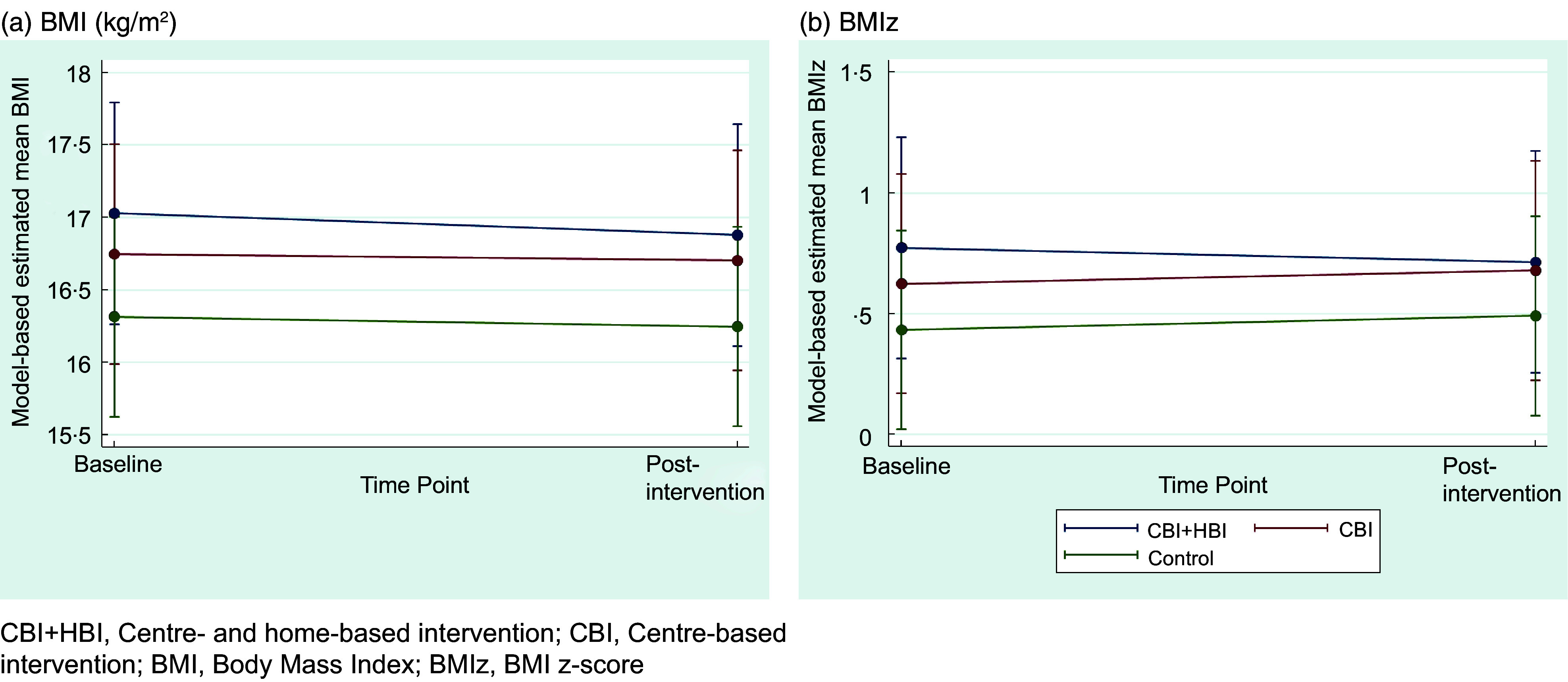
Model-based estimated mean outcomes of interest and corresponding 95 % CI

For the primary outcome (Table 4 and Fig. 2), there was no significant between-group difference in BMI at post-test but a significant within-group reduction in BMI (mean change (se) = –0·15(0·07), *P* = 0·04) in the centre + home-based intervention adjusting for outcome-specific significant confounders. There was also a significant within-group reduction in WAZ (mean change (se) = –0·07(0·03); *P* = 0·04) in the centre + home-based intervention children. Between-group differences in weight outcomes were found between children in the centre + home-based intervention and control children, though not all reached significance. Children in the centre + home-based intervention had a larger non-significant reduction in BMIz (adjusted difference (95 % CI) = –0·12 (–0·24, 0·01), *P* = 0·06) and BMI %ile (adjusted difference (95 % CI) = –3·27 (–6·67, 0·13), *P* = 0·06) compared with control children. However, centre + home-based intervention children exhibited a significant reduction in WAZ (adjusted difference (95 % CI) = –0·09 (–0·17, –0·002), *P* = 0·04) compared with control children.

**Table 4:** Adjusted change in outcomes of interest‡

Outcomes	Centre + home-based intervention (*n* 100)		Centre-based intervention (*n* 102)		Control (*n* 123)		Difference (centre + home-based intervention – control)			Difference (centre-based intervention – control)		
	Mean change	se	Mean change	se	Mean change	se	Difference	95 % CI	*P*-value	Difference	95 % CI	*P*-value
Weight gain outcomes												
BMI§	−0·15	0·07†	−0·04	0·07	−0·07	0·06	−0·08	–0·27, 0·11	0·40	0·03	–0·16, 0·21	0·80
BMIz||	−0·06	0·05	0·06	0·05	0·06	0·04	−0·12	–0·24, 0·01	0·06*	0	–0·12, 0·12	0·96
BMI %ile¶	−1·00	1·30	2·74	1·27†	2·27	1·14†	−3·27	–6·67, 0·13	0·06*	0·47	–2·89, 3·83	0·78
WAZ**	−0·07	0·03†	−0·03	0·03	0·02	0·03	−0·09	–0·17, –0·002	0·04†	−0·05	–0·13, 0·04	0·27

BMIz, BMI z-score; WAZ, body weight z-score.

* 0·05 <= *P* < 0·1.

† *P* < 0·05

‡ All models take into account the correlations between multiple measures from the same child and multiple children from the same centre and adjust for treatment, time, treatment × time, centre size, and outcome-specific significant confounding variables as noted below.

§ Based on a linear mixed effects model of 562 observations (average observations per child = 2, average children per centre = 46·8) adjusting for baseline height and change in height; ICC = 0·94 for measures nested within children; ICC = 0·003 for children nested within centres.

|| Based on a linear mixed effects model of 562 observations (average observations per child = 2, average children per centre = 46·8) adjusting for baseline height and change in height; ICC = 0·93 for measures nested within children; ICC = 0·003 for children nested within centres.

¶ Based on a linear mixed effects model of 562 observations (average observations per child = 2, average children per centre = 46·8) adjusting for baseline height and change in height; ICC = 0·91 for measures nested within children; ICC = 0·02 for children nested within centres.

** Based on a linear mixed effects model of 562 observations (average observations per child = 2, average children per centre = 46·8) adjusting for baseline age, baseline height and change in height; ICC = 0·94 for measures nested within children; ICC = 0·02 for children nested within centres.

## Discussion

To our knowledge, *¡Míranos!* is the first comprehensive obesity prevention intervention in the USA to target multiple EBRB in primarily Latino children from low-income families in organised childcare. Reducing excessive gain of adiposity in young children regardless of their obesity status can reduce the risk for early onset of obesity in children and adolescents and metabolic diseases in later life^(27)^. Although BMI in the centre + home-based intervention declined significantly from baseline to post-test, there was no significant difference in BMI change between the centre + home-based intervention and control or the centre-based intervention and control at post-test. Among additional measures of weight gain, the between-group difference in BMIz and BMI %ile approached significance, while WAZ was significantly different between the centre + home-based intervention and control.

The current study’s findings are consistent with results from recent RCT in childcare settings that did not demonstrate a robust effect in controlling excessive weight gain^(10,28)^. A 2020 Cochrane Review revealed that implementing evidence-based centre policies, practices or programmes was insufficient to affect child weight status, diet and PA in organised childcare, regardless of the level of implementation fidelity^(10)^. An RCT conducted in Head Start also showed that adding an obesity curriculum alone did not significantly reduce children’s level of obesity^(29)^. Although *¡Míranos!* was not efficacious in impacting BMI, especially in children in the centre-based intervention, the findings should be interpreted in consideration of secular changes in society, including Head Start, and issues in research methodology. Starting in 2017, Head Start implemented major changes in PA and nutrition policies in the Head Start Performance Standards^(13)^ that improved opportunities for daily PA, offered drinking water throughout the day, improved nutrition standards in meals and snacks, and incorporated culturally appropriate food services^(30)^. The implementation of the mandated changes in control centres might have diminished the effect of intervention goals and activities in the centre-based intervention developed before 2017 but could not be changed due to costs and resource demands^(31)^. Furthermore, some of the PA and nutrition policies and practices adopted in *¡Míranos!* are deemed obsolete according to new guidelines for PA^(32)^ and diet^(33)^. Future studies should incorporate the new recommendations and reexamine the efficacy of a childcare-based approach to prevent obesity in young children. Although our study was not powered to test differences between the centre-based intervention and the centre + home-based intervention, the favourable outcomes in the centre + home-based intervention support the importance of targeting multiple settings, i.e. the centre and home environment, in obesity prevention^(9)^.

From a design perspective, *¡Míranos!* was a complex intervention that incorporated a large number of evidence-based strategies to target barriers of multiple EBRB at the centre and home. However, it was not clear if the components of the multiple-level intervention generated a synergistic effect. In addition, feedback from Head Start staff revealed that the complicated delivery schedule might have overburdened Head Start staff, while various intervention components could have had counterproductive effects.^(34)^ Therefore, we plan to examine the effect of implementation fidelity on study outcomes and identify barriers to the delivery of *¡Míranos!*. Finally, future research should use a multiphase study design to examine optimal combinations of the strategies to improve intervention efficiency and management before large-scale deployment^(35)^.

The accelerated height gain among control children may have masked the favourable impact on BMI even though children in the centre-based intervention and the centre + home-based intervention gained less weight^(36)^. The higher velocity of height gain (i.e. being taller for age) indicates a faster growth rate in young children^(26)^ and is associated with a higher level of adiposity measures^(24,37)^. Among participants in the Hip Hop to Health Jr. studies, children in the intervention group gained more height and a lesser extent of weight compared with control children which led to a significant reduction in BMI in the cohort of African American children^(12,38)^. It should also be noted that the increase in HAZ was fastest and largest in obese compared with normal-weight Latino children aged 2–5 years enrolled in the Special Supplemental Nutrition Program for Women, Infants and Children in Los Angeles County, California^(39)^.

Another methodological issue was related to the discrepancies in study outcomes, a conundrum of using BMI in evaluating obesity interventions in children^(22,36)^. Although a change in BMI is the commonly accepted measure of excessive weight gain in youth populations in earlier years^(22)^, recent research demonstrates that a change in standardised weight measures such as BMIz was more predictive of changes in adiposity^(24,40)^ and cardiometabolic risk indicators^(41)^ in younger children. Other studies preferred BMIz when BMI did not change but height increased significantly in children^(41)^. Since excessive weight gain from BMI and BMIz are strongly correlated with adiposity in various degrees, the lack of significant between-group change in BMI should not overshadow the favourable changes in BMIz and WAZ among children in *¡Míranos!* centre + home-based intervention.

Did the lack of robust effect on children’s obesity outcomes in *¡Míranos!* weaken the support of a comprehensive environmental multi-setting approach in childhood obesity prevention? The improvement in BMI (–0·08, (95 % CI –0·27, –0·11)), BMIz (–0·12 (95 % CI –0·24, –0·01)) and BMI %ile (–3·27 (95 % CI –6·67, 0·13)) among the centre + home-based intervention children approached or exceeded the level of changes reported in two successful comprehensive interventions with a family component, the Children’s Healthy Living Program (percent of children overweight or obese –3·95 % (95 % CI –7·47, –0·43) and BMIz –0·06 (95 % CI –0·14, 0·03))^(42)^ and Australian Romp & Chomp (percent of children overweight or obese –2·7 %; BMI 0·004 (95 % CI –0·09, 0·09), and BMIz 0·01 (95 % CI –0·05, 0·07)).^(43)^ These programmes were implemented with low-income multiethnic young children and offered increased access to and support for PA and healthy eating within the children’s communities. While *¡Míranos!* modified the many relevant aspects of the centre environment (i.e. policies, social-cultural norms, programme resources and provision of small play equipment) and offered parents training and support in modifying the home environment, some important social, physical and financial barriers impacting EBRB in children at the centre and home are not adequately addressed^(44)^. Such barriers included the lack of developmentally appropriate playgrounds at the centres, limited access to play space and equipment in the child’s home or community, insufficient time for parents to play with their children due to excess work commitments, low affordability for and access to fresh fruits, vegetables, and nutritious food, and overexposure to low-quality processed foods and sugar-sweetened beverages, all of which have been linked to childhood obesity in low-income minority children in the USA^(45)^. While policy changes, education and training can be effective in curbing obesity among populations of higher income and educational achievement^(46)^, obesity prevention programmes that do not address inequity and inequality experienced by low-income families (e.g. purchasing power for and access to healthy food, availability of safe playgrounds or community parks) may be insufficient to significantly modify children’s EBRB^(44–46)^. Therefore, we speculate that the absence of community-based health improvement strategies to increase access to and support for healthy options and resources may explain the weakened impact of *¡Míranos!* ^(4)^. Future studies must address the disparities in access and resources related to social disadvantages and social determinants of health to increase the likelihood for children from low-income families to achieve equitable health outcomes^(47)^.

### Strengths and limitations

The commitment and support of local Head Start staff, leadership and parents were critical to the success of *¡Míranos!* treatment randomisation, intervention implementation, and study evaluation, and reflects Head Start’s commitment to children’s health^(48)^. Additionally, the intervention was developed and refined through several pilot studies leading up to the full trial that facilitated the formulation and tailoring of intervention and assessment protocols to be consistent with Head Start Performance Standards and infrastructure and acceptable for Head Start staff and parents.

There are several limitations to the generalisability and interpretation of these findings. The COVID-19 pandemic resulted in the loss of Cohort 2 participants, reducing the study sample and potentially impacting the statistical power to detect an intervention effect at 8 months post-test. Also, Head Start staff and data collectors were not blinded to the study centre condition, potentially limiting the generalisability of the study findings. Moreover, since we used a parallel RCT design, the effect of different components (e.g. the centre + home-based intervention *v*. the centre-based intervention) and specific programme elements (e.g. food tastings, staff wellness programme and home visits) were not evaluated. In addition, offering the I Am Moving, I Am Learning curriculum the literacy programme to control centres may have attenuated the effect of *¡Míranos!* ^(49)^. Finally, BMI is not sensitive to PA-induced changes in body composition (e.g. fat mass and bone density)^(36)^. Measures estimating a change in fat and lean mass, such as bioimpedance, should be used in the future^(50)^.

## Conclusion

Obesity research expert, Dr. Shiriki Kumanyika, Drexel University, has eloquently argued that the success of evidence-based obesity prevention strategies for low-income families must be accompanied by efforts related to ‘improving health options, economic and other resources, building community capacity, and decreasing deterrents to healthy behaviours in circumstances of systematic social disadvantage (page 9)’^(47)^. Although *¡Míranos!* failed to significantly reduce the excessive gain in BMI in predominantly Latino children from low-income families, examination of the plausible causes and favourable outcomes in children receiving the centre + home-based intervention offer directions in future studies to disentangle methodological challenges (i.e. secular trends and measurement of obesity) as well as to advance an agenda for a health equity-oriented obesity prevention approach.

## Supplementary material

For supplementary material accompanying this paper visit https://doi.org/10.1017/S1368980022002439. click here to view supplementary material
