# Supplementary material for: ¡Míranos! a comprehensive preschool obesity prevention programme in low-income Latino children: 1-year results of a clustered randomised controlled trial
Source: Public Health Nutr. 2022 Nov 11;26(2):476–87. doi: 10.1017/S1368980022002439 (PMC10172390; doi:10.1017/S1368980022002439)
Supplement: Supplementary file 1 [file S1368980022002439sup001.docx]

**Supplement Table 1. *¡Míranos!* Intervention Treatment Conditions and Objectives**

| Treatment | Treatment Component | Objectives |
| --- | --- | --- |
| Center-based Intervention | Physical activity and nutrition policy and environment | 1. Educate children to develop healthy habits for life  2. Offer 90-minutes free and teacher-led physical activity to children at the Center every day  3. Offer balanced healthy meals and snacks utilizing the USDA Child and Adult Care Food Program best practice recommendations |
|  | ¡Míranos! physical activity/gross motor program | 1. Children will be provided at least 90 minutes of structured and unstructured playtime each school day. 2. Children will participate in morning outdoor play (structured activity 15 min and free play 15 min) each school day. 3. Children will participate in active learning classroom activities during center time, transition, and breaks (30 min) each school day. 4. Children will be provided with outdoor play (structured activity 15 min and free play 15 min) each school day. (did it need to be in the afternoon?) 5. Screen time for entertainment at the center will be limited to 30 min a week. 6. Children’s sitting time will be <15 min in any setting except nap and mealtime. |
|  | Supplemental health education activities | 1. Utilize health education activities from Healthy Habits for Life from Sesame Street in daily lessons 2. Provide weekly health challenges 3. Offer food tastings (any specifics on frequency – like the others?) |
|  | ¡Míranos! staff wellness program | 1. Engage in healthy lifestyle habit changes through staff wellness challenges. |
|  | Meal pattern modification | 1. Increase the consumption of fresh fruit, vegetable, and whole grain at lunch and snacks, and reduce the consumption of sugar 2. Increase children’s exposure to healthy food |
|  | Staff development and training | 1. Review ¡Míranos! intervention plan and policies. |
| Home-based Intervention | Peer-led obesity education | 1. Change parental obesity-related beliefs and practices 2. Teach parents recommendations on preschool children’s body weight, PA, nutrition |
|  | Peer educator training | 1. Train the peer parent educators to deliver the wall poster parent education sessions |
|  | Home visits | 1. Develop skills and strategies for parents to promote PA, healthy eating, and sleep for their children at home 2. Encourage parents to be positive role models of healthy behaviors in the home |
| Control | ”I Am Moving, I Am Learning” obesity prevention curriculum | 1. Promote physical activity and healthy eating 2. Keep active control centers engaged in the study |
|  | Literacy Education | 1. Teach skills to parents on how to introduce basic literacy and nutrition concepts at home 2. Keep parents engaged in the study |
